# Pathological manifestations of granulomatous lobular mastitis

**DOI:** 10.3389/fmed.2024.1326587

**Published:** 2024-02-02

**Authors:** Leyin Cui, Chenping Sun, Jierong Guo, Xuliu Zhang, Sheng Liu

**Affiliations:** ^1^Department of Breast Surgery, Longhua Hospital Shanghai University of Traditional Chinese Medicine, Shanghai, China; ^2^Graduate School, Shanghai University of Traditional Chinese Medicine, Shanghai, China

**Keywords:** granulomatous lobular mastitis (GLM), cystic neutrophil granulomatous mastitis (CNGM), pathology, *Corynebacterium*, differential diagnosis

## Abstract

Granulomatous lobular mastitis (GLM) is a rare inflammatory breast disease with unknown etiology, characterized by non-caseous granulomatous inflammation of the lobules, which infiltrate lymphocytes, neutrophils, plasma cells, monocytes, and eosinophils may accompany. GLM is often misdiagnosed as breast cancer due to the lack of specificity in clinical and imaging examinations, and therefore histopathology is the main basis for confirming the diagnosis. This review provides an overview of the pathological features of granulomatous lobular mastitis and cystic neutrophil granulomatous mastitis (CNGM, a pathologic subtype of GLM). As well as pathologic manifestations of other breast diseases that need to be differentiated from granulomatous lobular mastitis such as breast tuberculosis, lymphocytic mastopathy/diabetic mastopathy, IgG4-related sclerosing mastitis (IgG4-RSM), nodular disease, Wegener’s granulomatosis, and plasma cell mastitis. Besides, discusses GLM and CNGM, GLM and breast cancer, emphasizing that their relationship deserves further in-depth exploration. The pathogenesis of GLM has not yet been clearly articulated and needs to be further explored, pathology enables direct observation of the microscopic manifestations of the disease and contributes to further investigation of the pathogenesis.

## 1 Introduction

**Figure 1:**
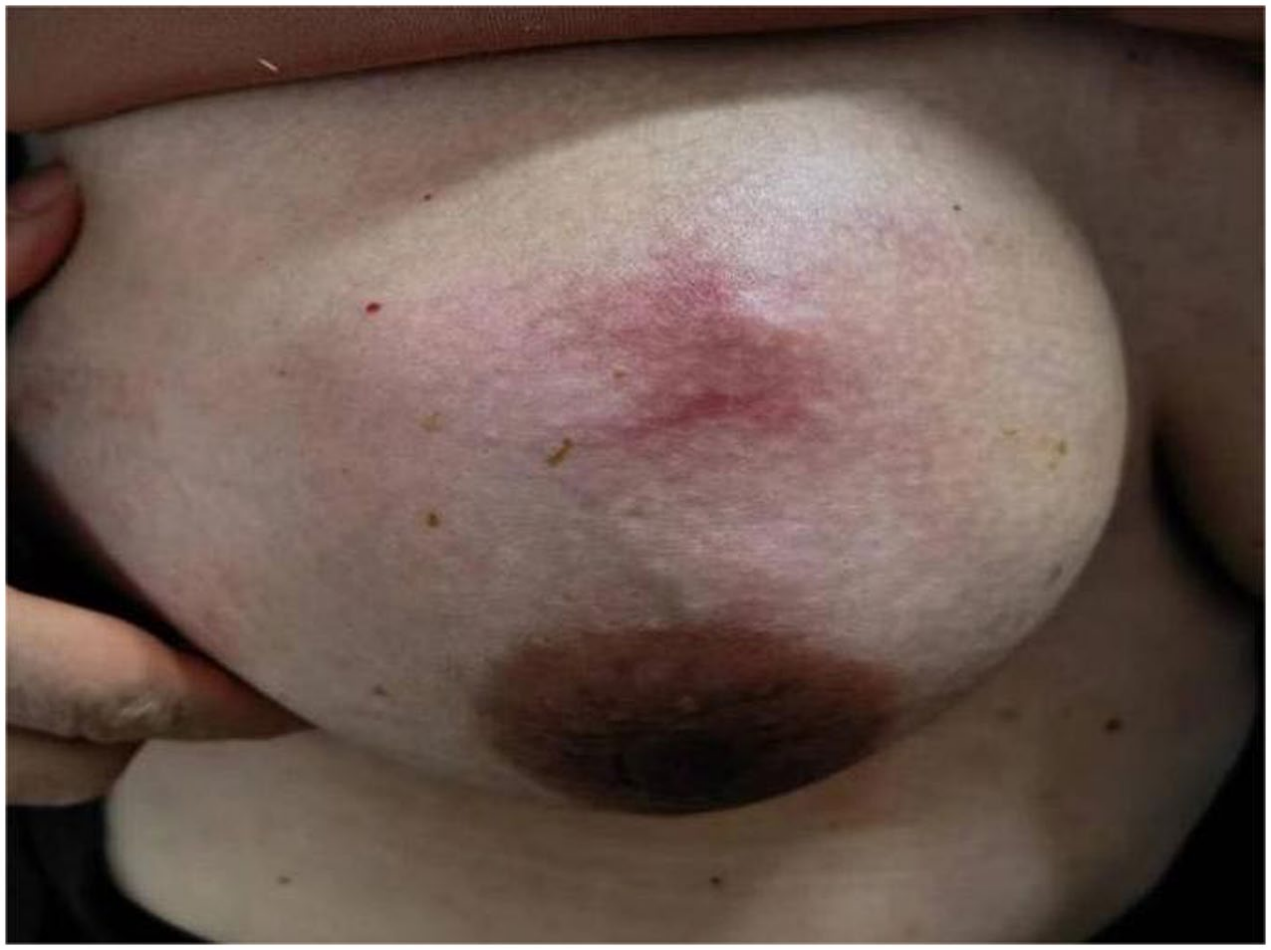
Painful lump in right breast with skin redness and swelling.

**Figure 2:**
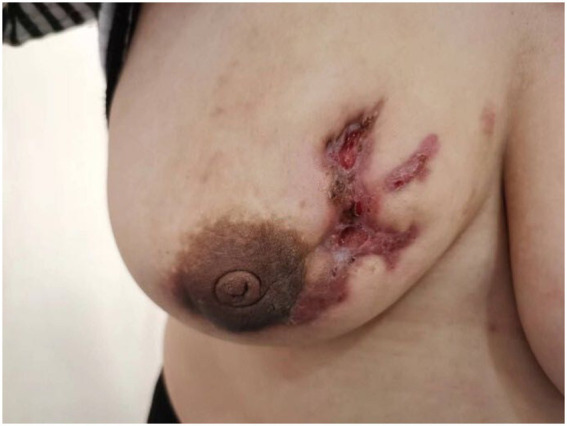
The skin of the right breast breakdown forms sinus tracts and ulcerated surfaces.

**Figure 3:**
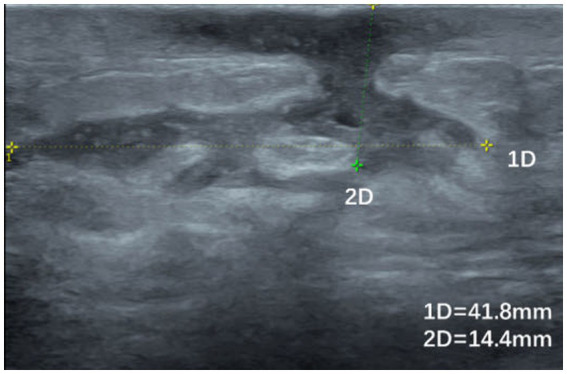
Ultrasound image of a patient with GLM with hypoechoic areas leading to the skin and sinus tract formation.

**Figure 4:**
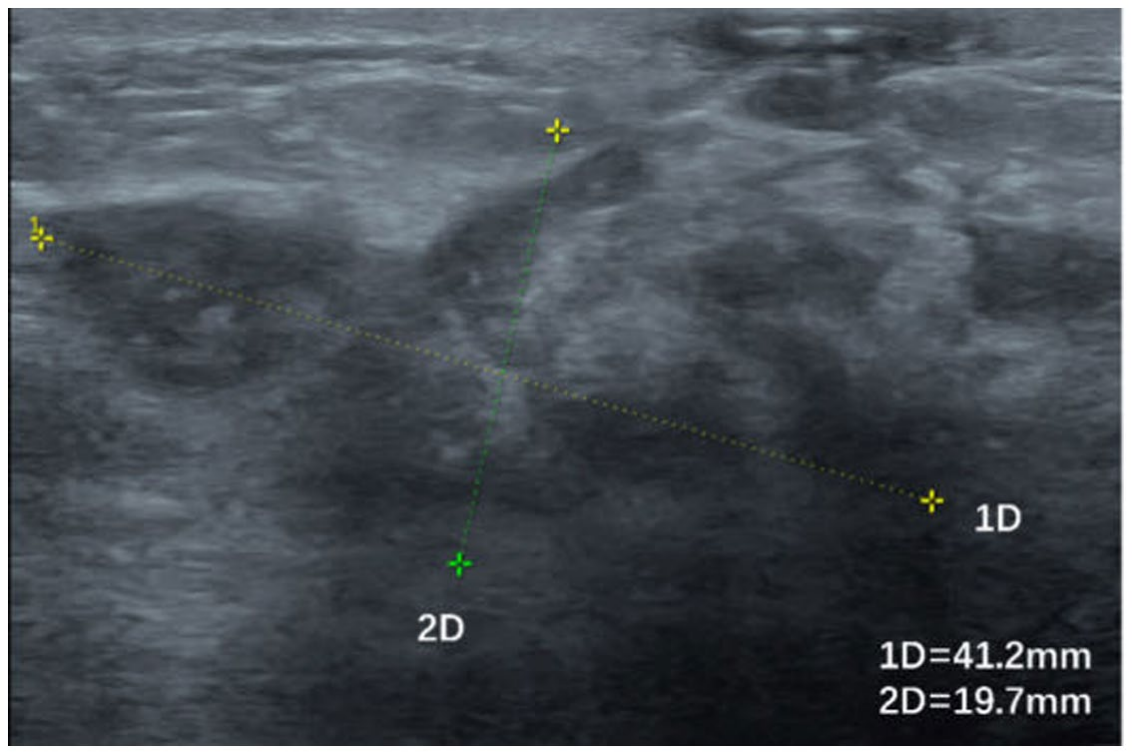
Irregularly shaped hypoechoic area with poorly defined borders and inhomogeneous internal echoes.

**Figure 5:**
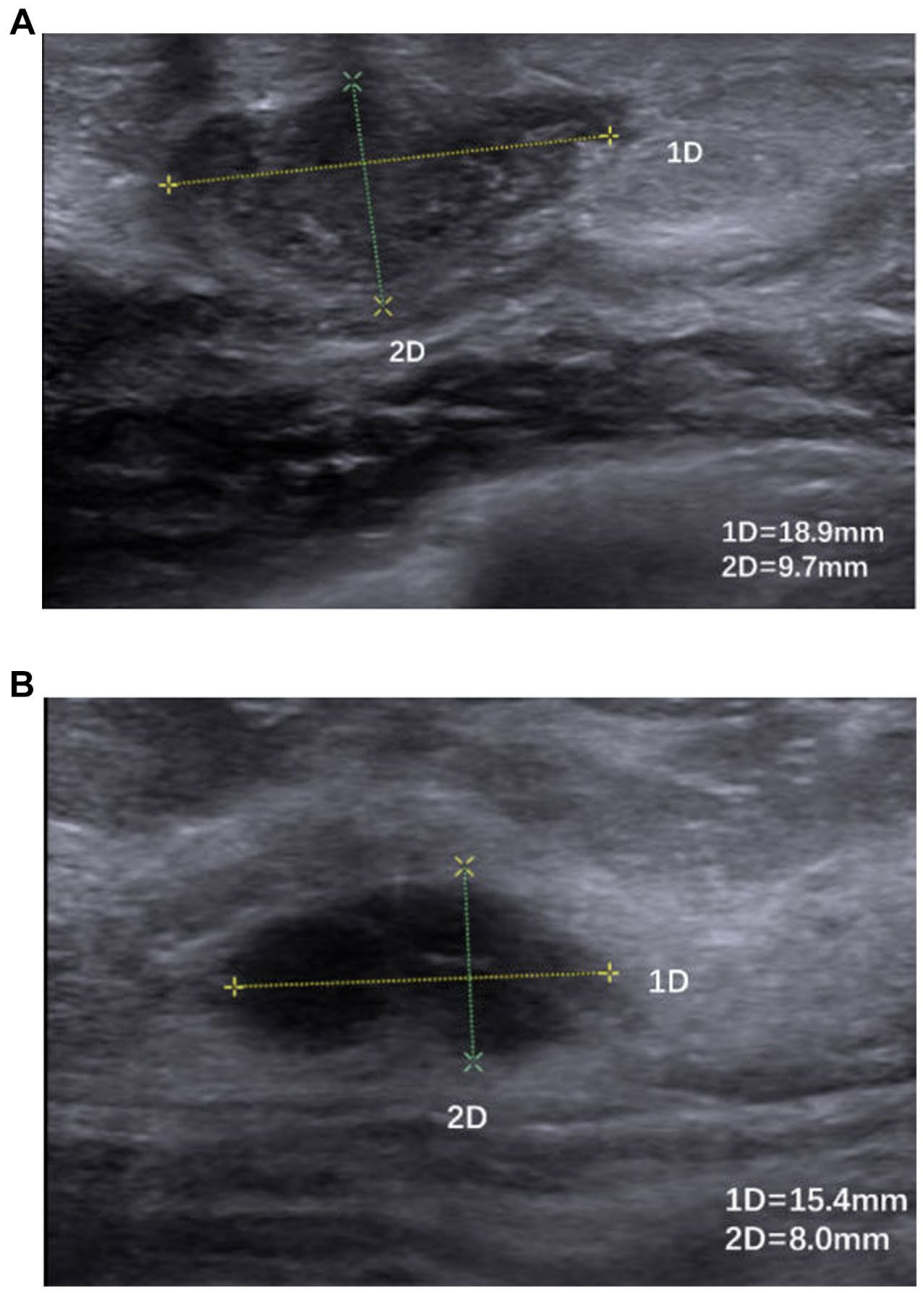
**(A,B)** Hypoechoic nodule with poorly defined borders, irregular morphology, and inhomogeneous internal echoes.

**Figure 6:**
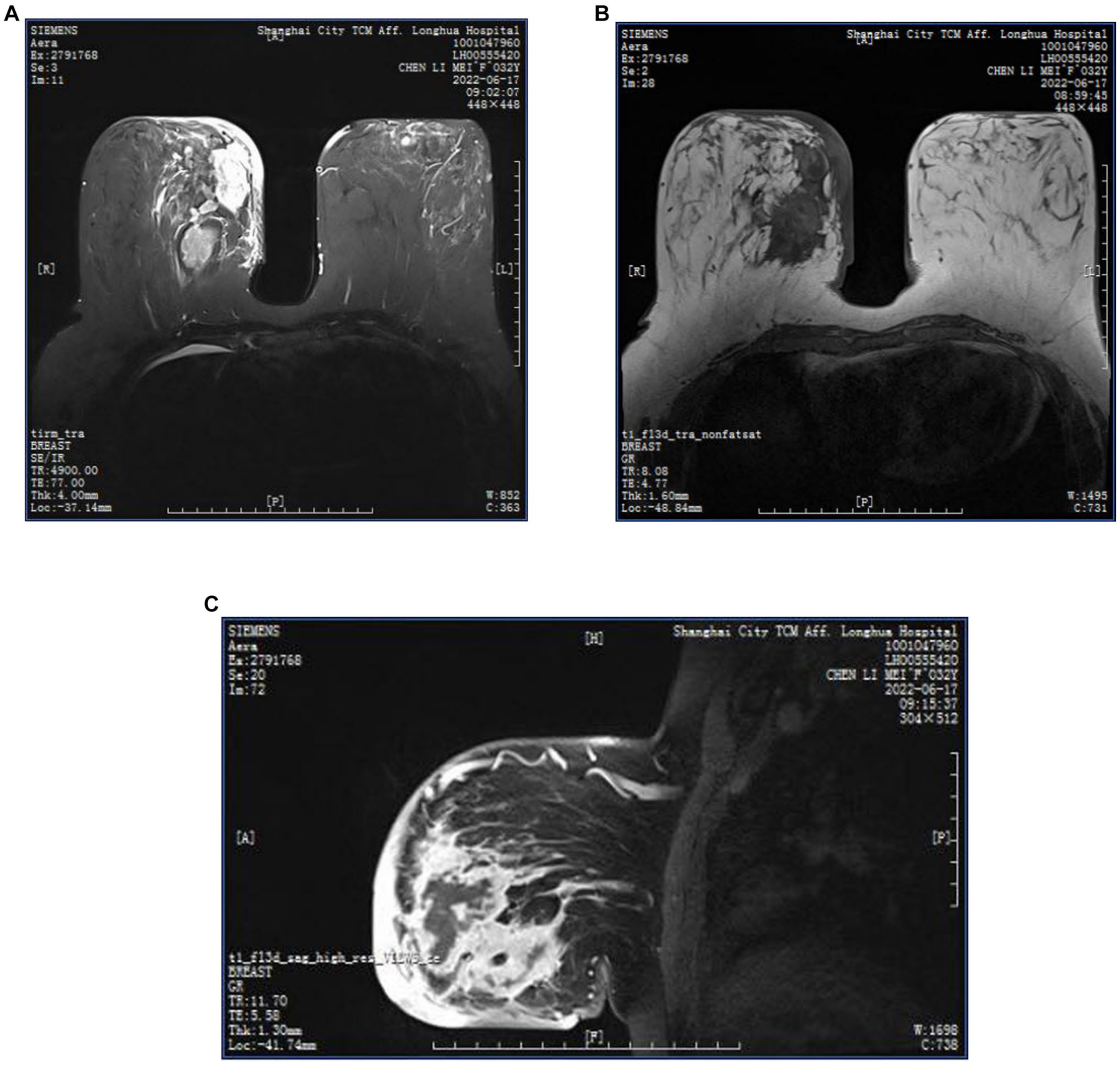
**(A–C)** MRI images of a GLM patient, multiple regionally distributed non-mass enhancing foci in the outer upper and inner upper quadrant of the left breast, inner upper and inner lower quadrant of the right breast, T1WI iso-slightly high signal, T2WI high signal, oedema around the foci, clusters of ring enhancement, thickening, and enhancement of the skin of the right inner breast, subcutaneous oedema. Multiple axillary lymph nodes on both sides, partially enlarged, with visible lymphatic gates.

Granulomatous lobular mastitis is a chronic benign inflammatory disease of the breast, predominantly observed in non-lactating women of reproductive age with a history of gestation and lactation (1, 2). More cases were reported in Asia and Mediterranean countries such as China and Turkey (3, 4). The etiology of granulomatous mastitis is still unclear and is currently thought to be related to immunity, bacterial infections, and hyperprolactinemia, besides, labor and lactation, trauma, oral contraceptive pill (OCP) and psychotropic use, alpha1-antitrypsin (AAT) deficiency, and type 2 diabetes mellitus are also thought to be associated with GLM, It may be due to physical or chemical stimuli increased ductal permeability and delayed-type hypersensitivity caused by spillage of secretions such as milk from the ductal lumen (2, 5, 6). GLM presents a unilateral (or occasionally bilateral) painful lump in the breast (Figure 1). Initially, the skin may be red or unchanged in color, but the lump gradually becomes septic, involving the skin and forming a deep sinus tract or ulcerated surface (Figure 2). Some patients may have sunken nipples and swollen axillary lymph nodes or have extramammary symptoms such as fever, joint pain, and nodular erythema of the lower limbs. Common sequelae include scarring, retraction of skin and nipple, and even shrinkage of the entire breast, which affects the quality of life in young women (7). The imaging presentation of granulomatous lobular mastitis is nonspecific. On ultrasound, it showed multiple irregular hypoechoic masses, tubular connections, angular margins, hyperechoic rim, internal vascularity, and fistulae, tiny flowing spots of light when an abscess forms (Figures 3–5) (8–10), could accompanied by skin thickening, subcutaneous edema, and reactive hyperplasia of axillary lymph nodes (11). MRI specifically shows peripherally enhancing fluid or solid masses (Figure 6) (12). Current treatment focuses on observation, application of antibiotics, steroids, immunosuppressants, surgery (13–16), and traditional Chinese medicine (17, 18), lack of standardized treatment protocols. Recurrence rates vary between treatments (5, 19, 20). PRL levels, overweight, FSH/LH, and *Corynebacterial* infection had an association with GLM recurrence (21, 22).

The fact that the diagnosis is almost based on pathology, the difficulty in treatment, prolonged disease duration, and the high recurrence rate make GLM a refractory benign disease. It is crucial to diagnose this disease correctly as it can lead to different treatment options, especially since it is difficult to differentiate GLM from breast cancer. Pathology as the gold standard for diagnosis, is the most intuitive and convenient way to access the microscopic manifestations of the disease. This narrative review provides an overview of the pathologic manifestations of GLM and common differential diagnoses, discussing the relationship between GLM and CNGM, and the relationship between GLM and breast cancer, exploring the pathogenesis of GLM in terms of bacterial infections and inflammation-cancer theories.

## 2 Methods of obtaining pathologic tissue

The three main methods of obtaining pathologic tissue in breast disease are fine needle aspiration, core needle biopsy, and surgery. Fine needle aspiration has an irreplaceable role in determining the benignity and malignancy of the disease and in obtaining bacterial culture material because of its convenience and low invasiveness (23), However, the amount of pathological tissue extracted is not sufficient and the presence of granulomas may be missed, or it may not be sufficient to determine whether the inflammation is mainly centered on the lobules of the breast (24). The core needle, although more tissue can be obtained than fine needle aspiration, is not sufficiently diagnostic (inflammation) (25) or difficult to distinguish from tuberculosis mastitis (TB) (26). In view of its convenience, histopathology obtained by core needle biopsy or surgery is clinically recommended as a diagnostic source.

## 3 Histopathological manifestations of GLM

### 3.1 Gross examination

The lesions seen in general are mostly ill-defined, tough or hard, a few are soft and hard, a few are brittle, irregularly solid or cystic in section, grayish white or grayish red or grayish yellow, with dark red or yellow corn-like nodules, scattered with multiple pus cavities/pitting foci of necrosis of varying sizes, and some describe the cystic area as decaying (27). The cystic areas contain grayish, grayish-yellow, or grayish-brown secretions, with sinus tract formation visible on the cut surface (25, 28–32). It has been described as a decaying cystic area containing gray, grayish-yellow, or gray-brown secretions, with sinus tract formation on the cut surface.

### 3.2 Microscopic presentation

#### 3.2.1 Typical microscopic presentation of GLM

**Figure 7:**
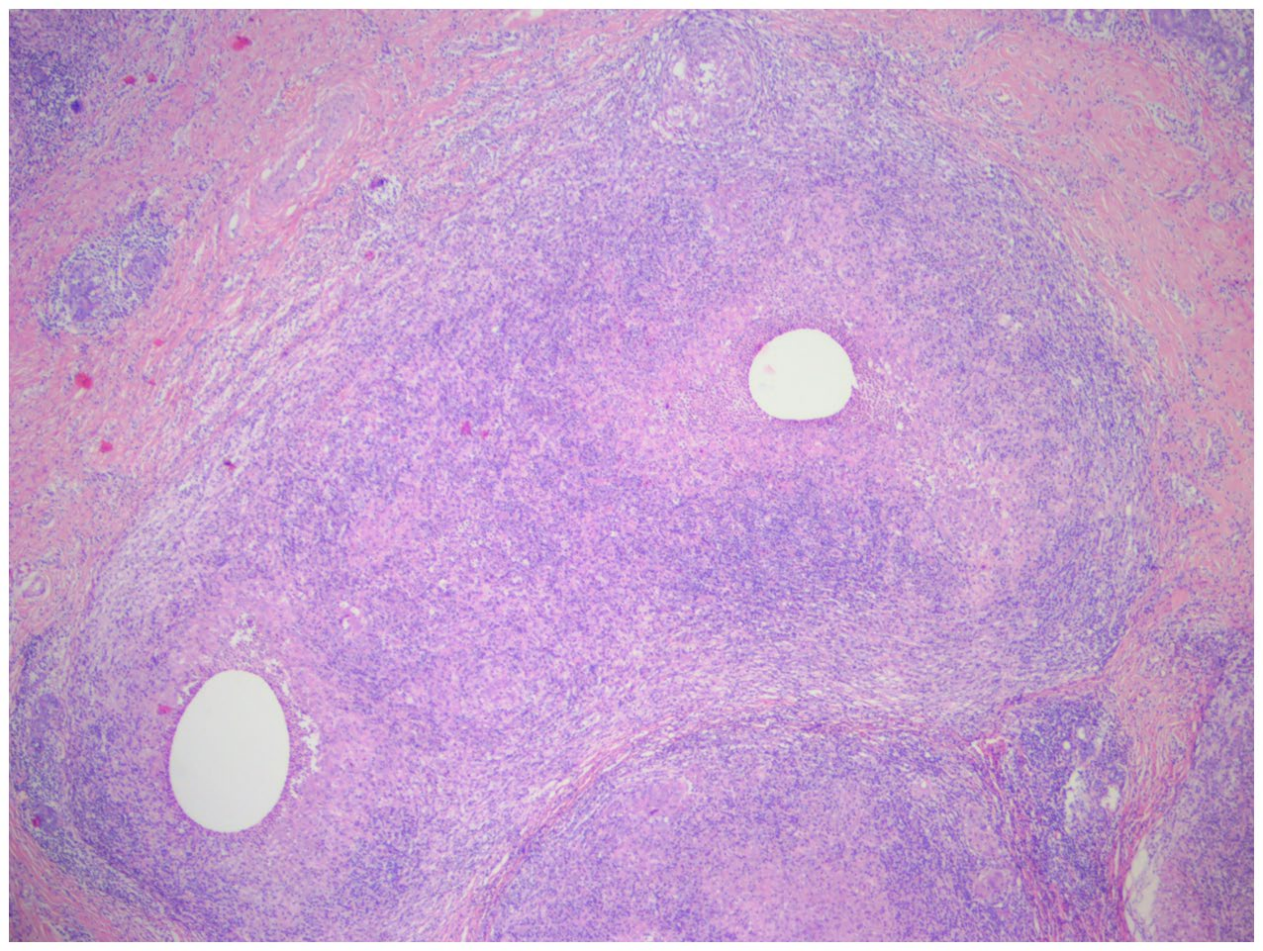
Two lesions with non-caseating necrotizing granulomatous lesions are partially fused, each with a vesicle visible in the center, and the normal tissue structure is disappearing. Visible epithelioid cells, and occasionally giant cells, acute and chronic inflammatory cell infiltration (lymphocytes, plasma cells, neutrophils) (H&E, ×40 magnification).

Microscopically, GLM is a non-caseating granulomatous inflammation of the ductal units of the lobules/terminal duct lobular unit, which may involve multiple lobules and may be associated with microabscesses (33, 34). The background is also infiltrated with inflammatory cells, mainly lymphocytes, neutrophils, plasma cells, monocytes, and eosinophils (Figure 7).

The lobular focus of granulomas can sometimes be masked and therefore makes the differential diagnosis difficult, mainly because (i) granulomatous tissue gradually replaces some or even all alveolar tissue, and lobular lesions are obscured and can fuse to form large lesions (35, 36). (ii) In the advanced stages, abscesses form, areas of necrosis expand, septic inflammation predominates and lobular structures fuse and disappear. (iii) Or small granulomas may form between lobules in the interstitium around the lesion, with a large number of microabscesses obscuring the granulomas, or even partially fused lesions with no visible granulomas (32). Tariq et al. pointed out that in the early stages of GLM, when suppuration is mild, the lobular structures are preserved in their entirety in the sections and are easily identifiable, which better defines classic GLM, but in the abscess phase the lobules gradually fuse, and in the late ulcerative stage the lobules fuse into a sheet, and in the more advanced stage the ducts are heavily necrotic and the lobular structures are eliminated, making them difficult to identify, with a small number of fibroblasts and neovascularisation visible, granulation tissue formation, and the formation of resorbing cystic vacuoles more easily seen than in the first two stages, with septic inflammation is predominant, creating a distinctive appearance of CNGM (28, 35, 37). Tariq et al. prefer that CNGM is a later stage of GLM rather than a subtype of GLM as most people believe. Associated with the close association of CNGM with *Corynebacterium*, this seems to point to the etiology of GLM as a bacterial infection.

Microabscesses are composed of neutrophils surrounded by epithelioid cells and monocytes and can be found in the lobular and ducts, in a few cases the entire epithelial lining of the duct is disrupted and replaced by inflammatory cells (38, 39). When larger abscesses or multiple small abscesses are formed, the lesions are more extensive and the inflammatory cell infiltration is more pronounced, involving the fat and skin, with skin breakdown (32, 35).

Multinucleated giant cells are predominantly Langhans-type giant cells with horseshoe-shaped nuclei, but a few foreign body giant cells can also be seen, and both can be present at the same time (23, 40). Ail DA believes that foreign body giant cells are more common in GLM and differentiates it from tuberculosis mastitis with a high number of Langhans-type giant cells (41). Ail DA suggests that foreign body giant cells are more common in GLM, and thus differentiates it from tuberculosis mastitis with more Langhans type giant cells. No phagocytosis in multinucleated giant cells (30). The presence of multinucleated giant cells in the ducts is sometimes associated with penetration (38). Eosinophilic infiltration is usually rare and variable in number (42, 43). Lacambra et al. found large numbers of eosinophil infiltrates, presumably related to the flow of protein secretions into the lobular stroma (44). Plasma cells are uncommon, accounting for essentially no more than 35%, and may be associated with mild to moderate lymphocytic vasculitis (40, 45, 46). Lymphocytic infiltration is common (47), and normal breast lobules also contain myeloid and lymphoid cells. In the study of Tse et al. (40), lymphocytes accounted for the majority of inflammatory cells (≥65%), with a lower proportion of neutrophils mostly accounting for less than 35%, and a large plasma cell infiltrate has also been seen (44, 48). In granulomatous lesions, neutrophil aggregation bands have more peripheral CD3+ lymphocytes than CD20+ lymphocytes (49). Anousha et al. suggest that granulomas, lobular central inflammation, and neutrophil infiltration are the pathological triad of GLM (50). This may be related to the different clinical staging of the obtained GLM tissue, with Yu et al. noting that the microscopic neutrophil count varies from the mass stage to the abscess stage to the post-ulcerative stage, and can vary from rare to numerous neutrophil infiltrates (28). Few researchers have described the pathological features of GLM according to clinical stage, so it is often difficult to detect microscopic patterns of GLM in terms of the way in which the various cell types are arranged and the changes in their proportions.

#### 3.2.2 Typical GLM accompaniment and merging

GLM is often associated with mammary duct ectasia (MDE), with inflammation in or around the ducts, but the inflammatory response is usually unremarkable. In the study by Ling Chen et al., the percentage of GLM merged with PDM reached 5.3%, they hypothesized that this was due to the involvement of the ducts by lesions in the lobules (49). The ducts are often lined with secretions, inflammatory exudates, exfoliated epithelium, and foamy histiocyte collections, with hyperplastic degeneration and focal exfoliation of the ductal epithelium, and fibrous thickening of the ductal wall, surrounded by a distinct lymphoplasmacytic infiltrate. The fusion lesions are mainly between GLM lesions, whereas fusion lesions between mammary duct ectasia and GLM are rare (51). There may also be subacute or chronic inflammation of the interstitium, combined fibromatous nodules/fibroadenomas, combined intraductal papillomas, combined fibrocystic changes/cystic hyperplasia, combined sclerosing adenopathy, cholesterol crystals and calcification (25, 43, 46). Metaplasia apocrine may be present (52). There may be mild fibrosis of the interlobular stroma surrounding the lesion (30). There may be mild fibrosis of the interlobular stroma surrounding the lesion. Fat necrosis may be seen (42, 50). Squamous epithelial metaplasia may occur in both lobules and ducts (23).

#### 3.2.3 Atypical microscopic presentation of GLM

Naik et al. suggest that one of the new features of CNGM is basophilic fibrous material surrounded by inflammatory cells and giant cells (38). This feature has not been described by others and its significance is unknown. Other uncommon features are caseous necrosis, marked eosinophil infiltration, non-granulomatous inflammation, and foamy histiocyte infiltration, granulation tissue formation (48, 50).

#### 3.2.4 GLM atypically presents as a combination of other diseases

Sometimes GLM is not isolated, Choi SH showed an example of an initial right breast lesion diagnosed as GLM and a secondary lesion in the opposite breast diagnosed as tuberculosis mastitis 5 months later, where the patient had a positive TBC-PCR (polymerase chain reaction for tuberculosis) and the mass completely disappeared after 12 months of anti-tuberculosis treatment (53). GLM can also be combined with atypical manifestations or/and malignancy, Çalış H reported a case of GM combined with breast cancer (54). Özşen M reported 2 cases of ductal carcinoma *in situ* associated with lesions detected in patients with recurrent GLM (52). Sometimes breast cancer can also cause inflammation and look like GLM (50, 54). This tells us to be careful in identifying GLM even when pathological tissue is obtained, and that as much breast tissue as possible will be better for a clear diagnosis, which is why we do not recommend fine needle aspiration.

## 4 Histopathological manifestations of CNGM

### 4.1 CNGM microscopic presentation

**Figure 8:**
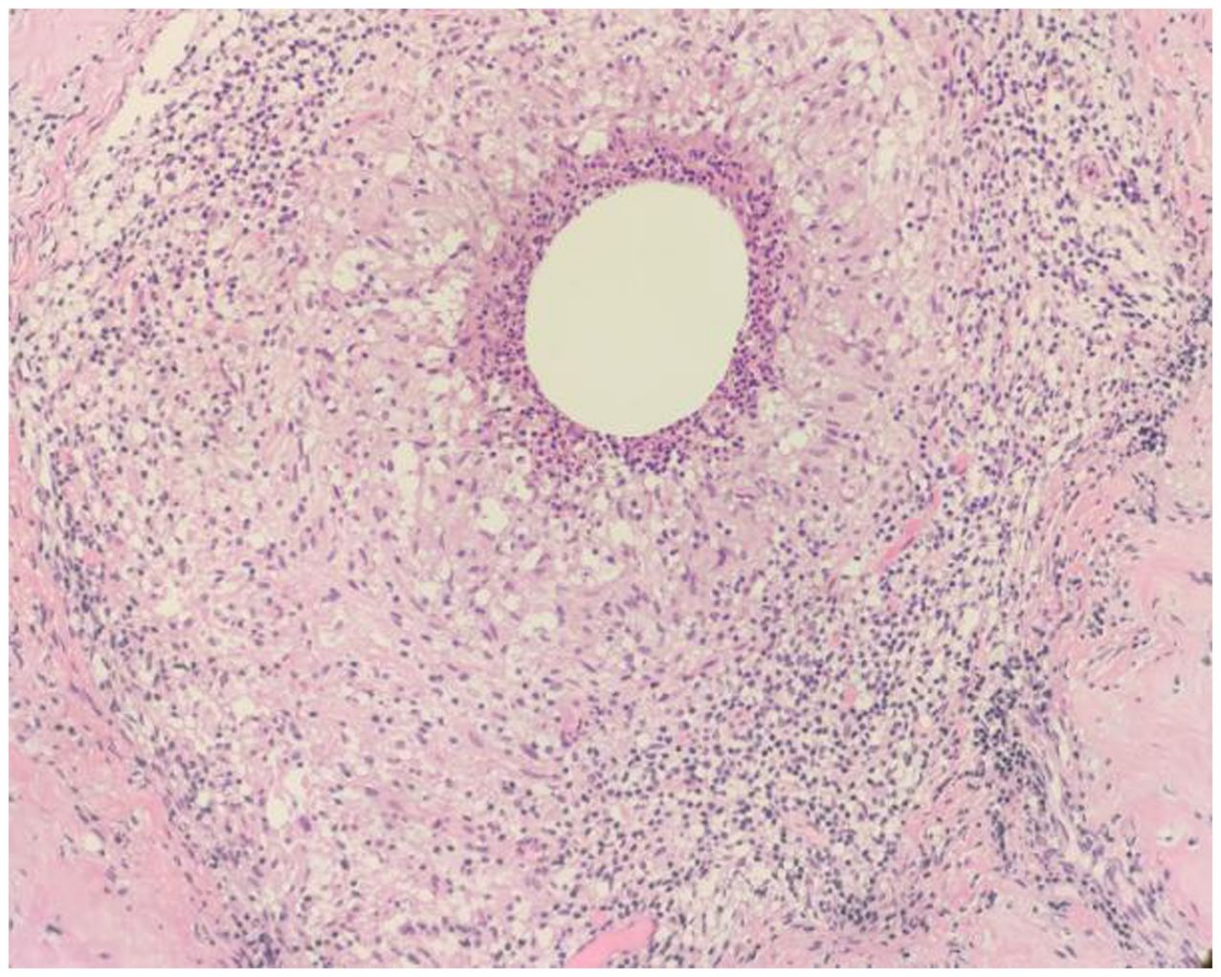
Examples of CNGM, granuloma formation from epithelioid cells, with a central vesicle surrounded by neutrophil accumulation. Infiltration of peripheral lymphocytes, plasma cells, neutrophils and a few eosinophils (H&E, ×200 magnification).

**Figure 9:**
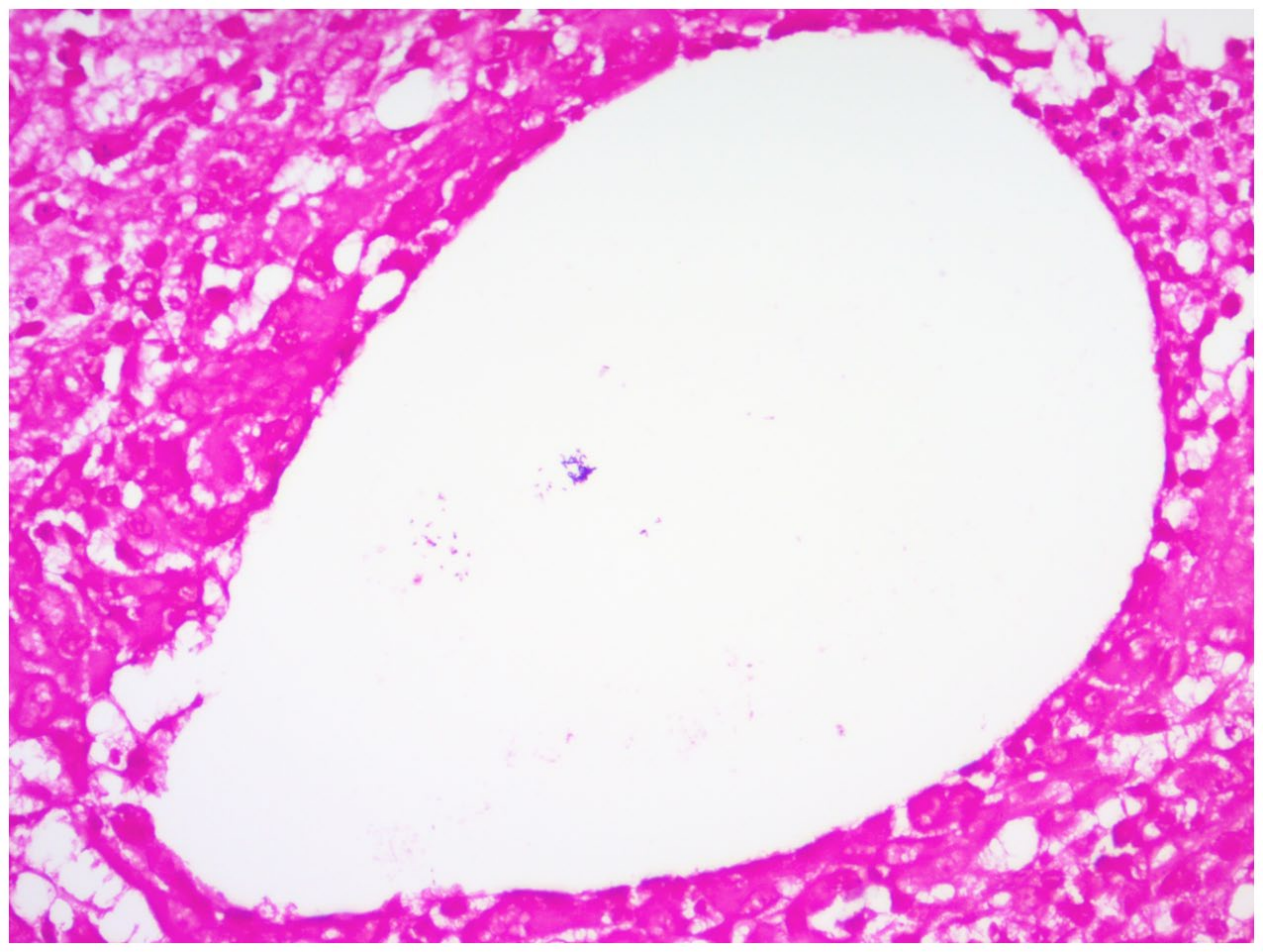
Gram-positive coryneform bacteria arranged in clusters stained purple can be seen in blank lipid vesicles (Gram stain, ×400 magnification).

**Figure 10:**
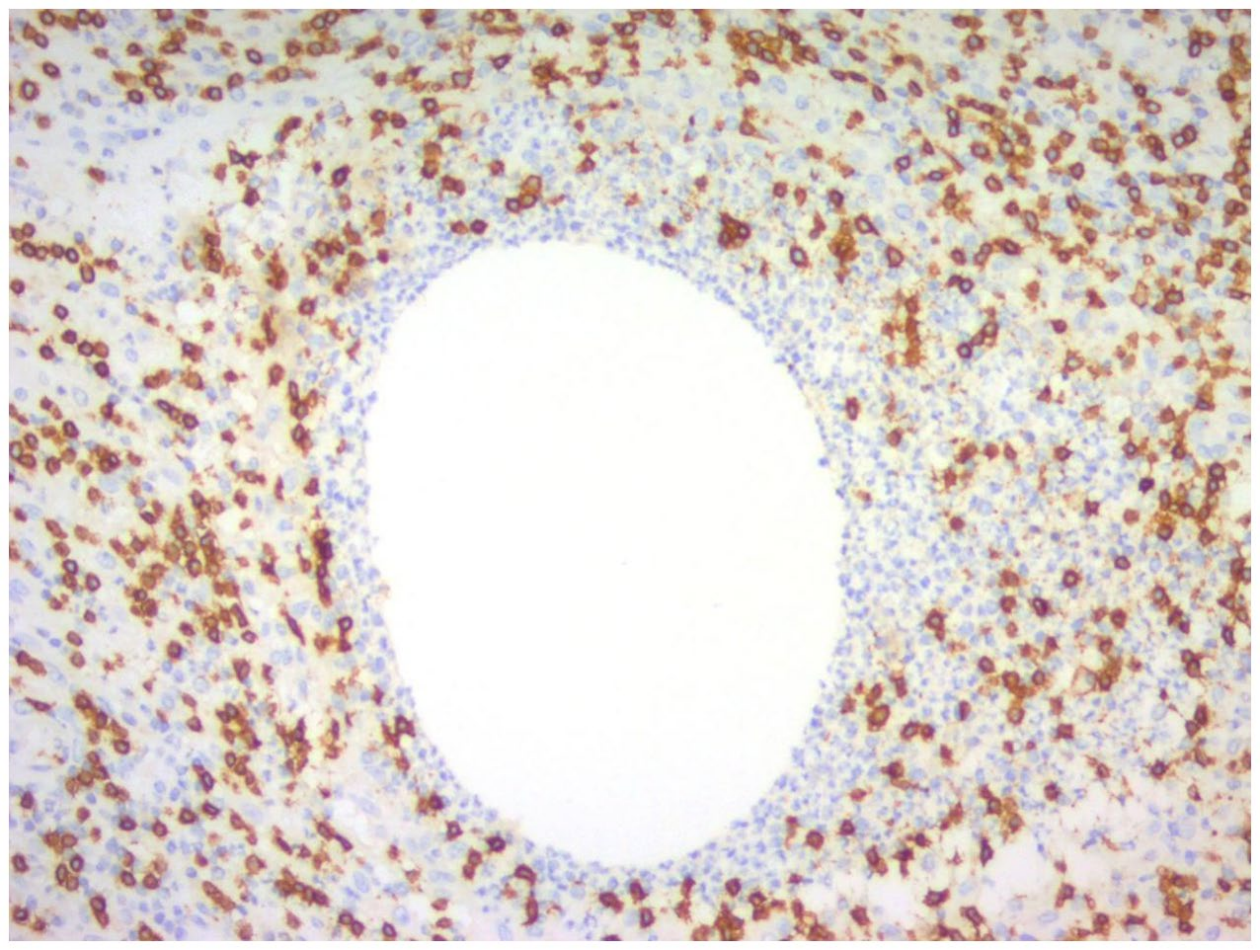
Immunohistochemical picture of CNGM with CD3-positive T lymphocytes stained brownish yellow and distributed at the periphery of the neutrophil aggregation band (En Vision, ×200 magnification).

**Figure 11:**
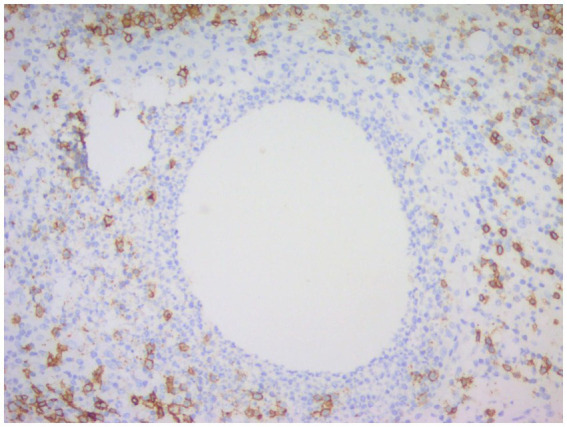
Immunohistochemical picture of CNGM, CD20-positive B lymphocytes were stained brownish yellow and distributed in the periphery of the neutrophil aggregation band, and their location was more peripheral compared to CD3-positive T lymphocytes (En Vision, ×200 magnification).

**Figure 12:**
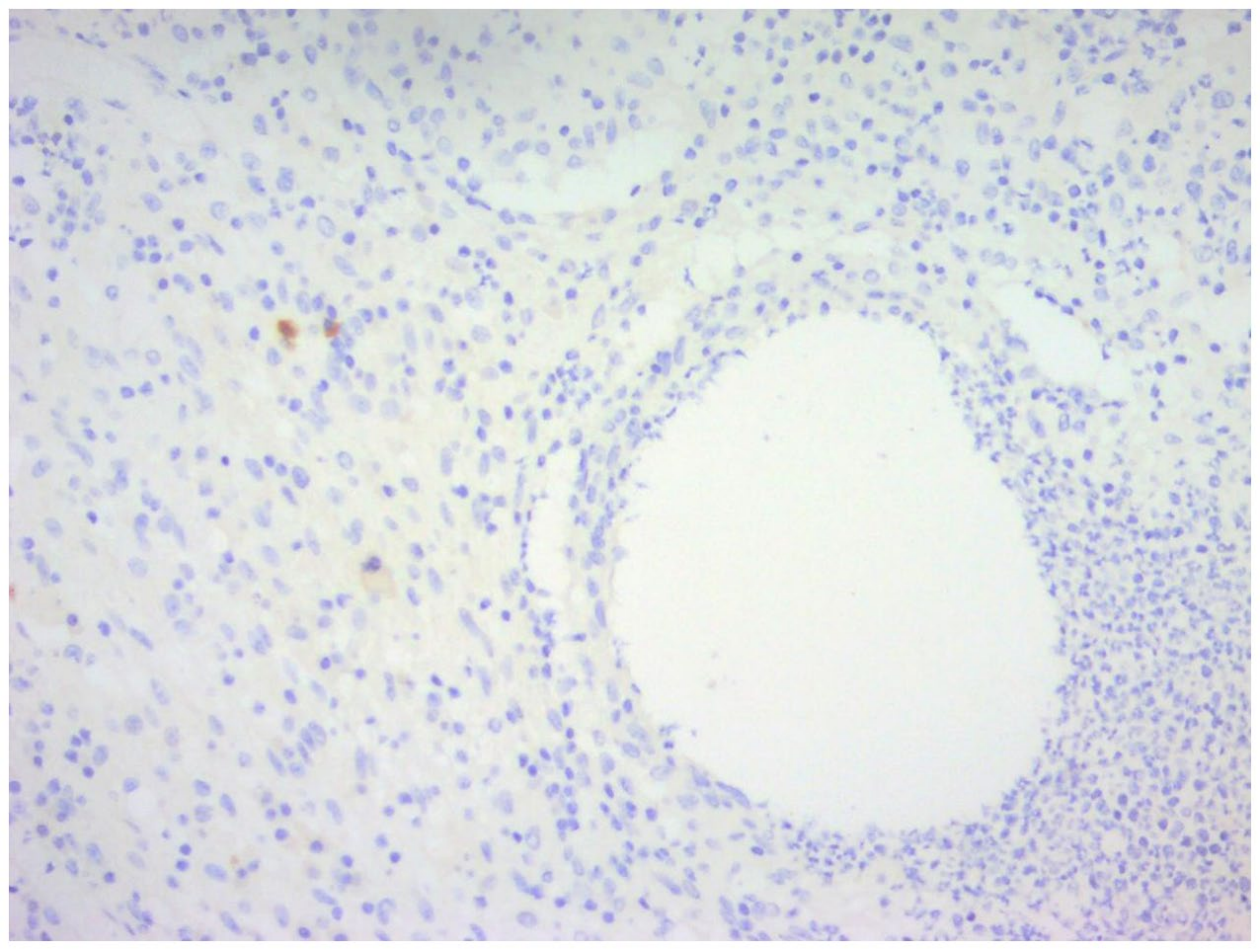
Immunohistochemical staining of CNGM, a few IgG4-positive plasma cells stained brownish-yellow can be seen around the vesicle (En Vision, ×200 magnification).

**Figure 13:**
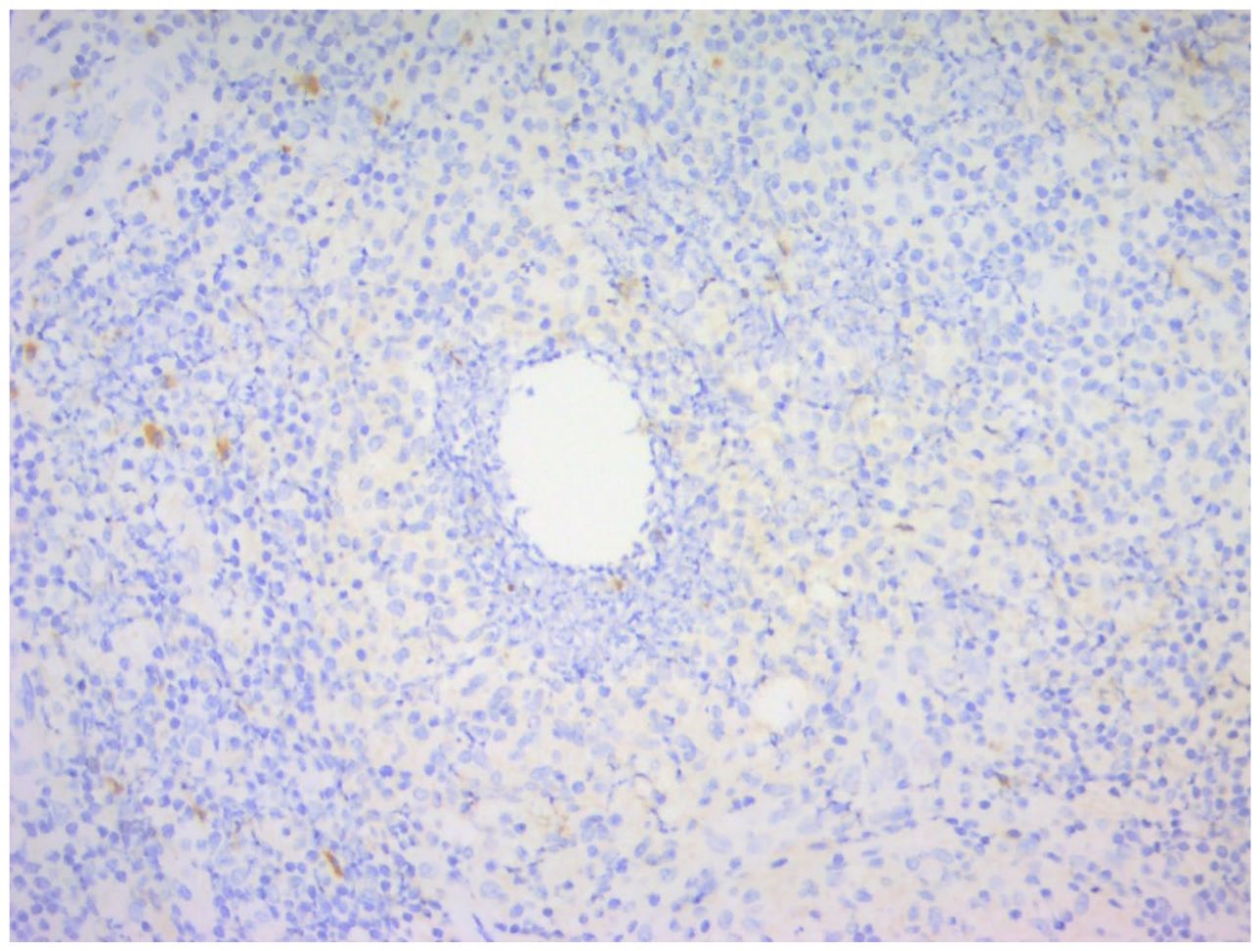
Immunohistochemical staining of CNGM, a few IgG-positive plasma cells stained brownish-yellow can be seen around the vesicles (En Vision, ×200 magnification).

CNGM is now considered a specific subtype of GLM with distinctive histopathological features, possibly associated with *Corynebacterium*, microscopically appears as a purulent lipid granuloma, surrounded by a central vacuole (cystic space), usually thought to be formed by lipolysis, with the size of several merged adipocytes (200–800 μm), surrounded by neutrophils, the thickness of the edge of which can be thin or thick (Figure 8). The number of neutrophils varies and the thickness of the rim surrounding the vesicle can be thin or thick, thus forming a microabscess in the granuloma, which is then surrounded by histiocytes, variable numbers of lymphocytes, plasma cells and Langhans giant cells, forming a definite granuloma (37, 55, 56). Sometimes granulomas can be poorly formed (57). However, not all granulomas have cystic vesicles, and neutrophilic inflammation and microabscesses can be seen outside the granuloma, and more often the granulomas fuse with each other to obscure the lobule-centered distribution characteristic of the granuloma (55). There are also cases where there are microorganisms in the lipid vacuoles surrounded by neutrophils but no granulomas. Gram-positive bacilli (GPB) are sometimes seen in the vesicles (Figure 9), apparently rod-shaped, and some of the bacilli are internally beaded or dendritic, arranged in a fenestrated pattern, forming wedge-shaped features (36, 45, 47). The microcystic vacuoles may sometimes be in clusters (38). Although GPBs are not always visible in the vesicles, they are confined to the interior of the lipid vacuoles in identifiable cases (24). Immunohistochemistry is increasingly used in the diagnosis and differential diagnosis of difficult breast lesions and is also the main means of molecular typing of breast cancer and screening of precise therapeutic markers, but in GLM and CNGM, in addition to identifying IgG4-related sclerosing mastitis (IgG4-RSM), its specific role is still unclear and lacks clinical significance (Figures 10–13).

### 4.2 *Corynebacterium* and CNGM

Gram-positive bacilli in lipid vacuoles are currently dominated by *Corynebacterium*, with the most common isolate being *C. kroppenstedtii*, followed by *C. amycolatum* and *C. tuberculostearicum* (50, 58). Other pathogens such as *P. oleovorans*, *human gammaherpesvirus 4*, *A. baumannii*, *T. thermophilus* are likely to be closely related to GLM (48). *Staphylococcus epidermidis*, *Staphylococcus aureus*, *Pseudomonas aeruginosa* etc. have also been cultured (59). There are also cases of mixed infections of *Corynebacterium* with other bacteria, or *Corynebacterium* is not the predominant pathogen (60, 61). The abundance of *Corynebacterium* in GLM patients showed inter-individual variability (62). The pus samples are more diverse than their skin samples (63) and are more abundant than in tissue samples. In a study by Wen Chen et al., the samples from 34.1% GLM patients had a *Corynebacterium* abundance of >1% (1.08–80.8%), with 53.3% displaying an abundance of >10% (64). Pathogen discovery will help guide clinical treatment. *Corynebacterium* was first isolated in 1998 from a human sputum sample (65). Lipophilic antibiotics may be more effective in treating *Corynebacterium*-associated breast infections, such as rifampicin, clarithromycin, and methotrexate-sulfamethoxazole (66). Other sensitive drugs such as vancomycin and gentamicin may also be used as adjunctive therapy (67, 68).

Positive bacterial cultures for *Corynebacterium* are most often seen in the abscess and refractory types (the two types are not statistically different) (35, 37). Patients with CNGM are younger, have larger masses, are more likely to be painful, febrile or with high neutrophils, form sinus tracts, and are more likely to recurrence (24, 69, 70). In the study by Tan QT et al. the risk of recurrence 2.64 times higher in patients with *Corynebacterium* infection (71). This may confirm the role of *Corynebacterium* in GLM in promoting abscess formation.

In fact, CNGM may be underdiagnosed because (i) Gram staining is usually limited to one or a few sections of formalin-fixed paraffin-embedded. (ii) Not all vesicles contain GPB. (iii) *Corynebacterium* are difficult to culture, whether the microbiological finding should be part of the diagnostic criteria remains debatable (72).

Based on nanopore sequencing and bacterial culture, Xin-Qian Li et al. find that the bacteria positive rate and vacuoles positive cases in the early stage (only hard mass) was significantly higher than that in late stage (medium/soft mass, with skin inflammation, abscess, fistulas or ulcers), the detection rate of bacteria in the early stage of GLM was over 80% and the dominant bacteria were *Corynebacterium* species (64%). Thus they speculate that *C. kroppenstedtii* may be initially present in the breast tissue at the early stage (73). Tariq H considers the early identification of *Corynebacterium* in deep breast tissue, causing peripheral granulomas and purulent inflammation, to be strong evidence of *Corynebacterium* pathogenicity (37).

However, the idea of *Corynebacterium* as the cause of GLM is still being questioned due to the negative microbiological cultures, the poor response to antibiotics, improvement of symptoms with cortisol or immunosuppressive drugs, the concomitant erythema nodosum of the lower limbs in some patients, and the self-limiting nature of GLM, which tends to be an autoimmune disease. And it remains controversial whether CNGM, as a pathological subtype of GLM, belongs to two different diseases from non-CNGM GLM, the relationship between GLM and CNGM needs further clarification.

Additionally, the classification of the period attributed to the patient’s disease in future research needs to be further refined, cause not all patients clinically undergo the process of abscess formation, and all patients with abscesses do not present it at the same period, in addition to the type of mass, the duration of the presence of the mass type, and the impact of treatment methods needs to be taken into account.

## 5 Techniques used in the diagnostic process of GLM, CNGM

### 5.1 Differential diagnosis of GLM from other diseases by staining

As an exclusionary disease, GLM is not associated with infectious diseases, other immune diseases, etc. Wu JM believes that once malignancy is excluded, infectious factors causing granulomatous mastitis should be considered and all cases should be subjected to special staining and microbiological cultures to exclude bacterial, fungal, parasitic and other sources of infection; especially in areas where tuberculosis is endemic, breast tuberculosis should be kept in doubt (72). In addition to the usual Gram stains the main ones are silver hexamine and Schiff periodate stains to exclude fungal infections, antacid stains (Wade-Fite, Ziehl-Neelsen), PCR tests, molecular tests for *Mycobacterium tuberculosis* to exclude *Mycobacterium tuberculosis*/Nontuberculosis mycobacteria infection (36, 57, 74, 75).

### 5.2 Methods for detecting *Corynebacterium*

*Corynebacterium* is generally detected by Gram staining, but its staining sensitivity is low in paraffin sections, and in a study by Tariq H et al., the Gram staining positivity of paraffin sections of *Corynebacterium* was significantly lower than that of PCR (17.9% vs. 68.7%) (76). Sangoi AR et al. found that cutting Gram-stained tissue sections of CNGM cases to a thickness of 6 μm instead of the traditional 4 μm increased the detection rate of Gram-positive bacilli (77). However, even with PCR testing, as only one section is selected per case, sampling may be falsely negative, and there is attrition of lesion tissue during processing, and dewaxing and DNA extraction may result in loss of cystic space contents, so Tariq H strongly recommends obtaining fresh biopsies for microbiological studies in suspected GLM cases, not only to improve PCR detection rates, but also to allow for culture. This would not only improve PCR detection but also allow antimicrobial susceptibility testing of positive cases, which would hopefully improve clinical outcomes with targeted antibiotics (76). Zhu Yongze et al. concluded that as long as a single *Corynebacterium* is isolated from a sterile site specimen in culture, it should be treated as pathogenic, even if no Gram-positive bacilli are seen in the original smear and only a certain number of leukocytes are present (58). The culture of *Corynebacterium* is now mostly done with blood plate, blood plate +1% Tween 80, extended incubation time and retention of tissue around the pus can increase the positive culture rate of *Corynebacterium* (59). The rate of positive culture of *Corynebacterium* can be increased by prolonging the culture time and leaving the tissue around the pus.

The more advanced methods for the identification of pathogenic organisms in CNGM include 16SrRNA gene sequencing, matrix-assisted laser-resolved ionization time-of-flight mass spectrometry (MALDI-TOF MS), rpoB gene sequence amplification by PCR, Sanger sequencing techniques (37, 38, 62, 67).

## 6 Differential diagnosis of GLM

### 6.1 Breast cancer

As the patient’s presentation is similar to breast cancer both clinically and on imaging, breast cancer becomes the primary diagnosis to be differentiated. The use of core needle biopsy or surgery to obtain pathological tissue is recommended. As the incidence of GLM has been increasing in recent years, cases of GLM in combination with breast cancer have gradually emerged. The diagnosis of breast cancer could be delayed because GLM has a long course and varied clinical presentation, its inflammatory features could have masked the presence of breast cancer until the clinical and imaging manifestations continued to suggest the risk of breast cancer or a new lump was found, which was then taken seriously by the clinician, lead to a new round of pathological biopsy (78–80).

Inflammation has now been progressively shown to be an important factor in tumor progression, chronic inflammatory responses promote cell division and repair, creating an environment that stimulates cancer growth and progression (81). Mammary ductal epithelial cells of GLM and plasma cell mastitis (PCM) showed injury and apoptosis, and MAC (C5b-9n) was mainly located on their cell membrane (82). Compared to healthy tissues, IGM tissues have elevated levels of both immune system and cancer-related proteins (83).

Currently, some large samples have shown that patients with mastitis have an increased risk of developing breast cancer and that mastitis can be considered a risk factor for breast cancer (84, 85). Further, respectively, observational research is needed on the relationship between lactational mastitis, GLM, and breast cancer, and specific mechanisms need to be explored.

### 6.2 Breast tuberculosis

There is a lot of histological overlap between GLM and tuberculosis, with granulomas and giant cells present, and it is particularly easy to misdiagnose on the basis of cytologic features alone, especially with a small number of fine needle punctures. Unlike the granulomas of GLM, which are centered on the lobules of the breast, the granulomas of TB distribution are irregular. The granulomas of GLM can be septic and necrotic, but TB is more prone to necrosis, especially the characteristic caseous necrosis (26). Ail DA et al. noted that Langhans giant cells are common in tuberculosis of the breast, whereas foreign body giant cells are common in GLM (41). However, most scholars have observed that Langhans cells are in fact not a minority in GLM (23, 40, 55). The predominance of neutrophils in the inflammatory infiltrate also contributes to the diagnosis of GLM (86). neutrophils and vesicles in TB are uncommon. Positive bacterial culture and Z-N staining is the gold standard for the diagnosis of TB, but the sensitivity is low and can be found by PCR for *Mycobacterium tuberculosis* (26, 41).

### 6.3 Lymphocytic mastopathy/diabetic mastopathy

Usually the patient has diabetes mellitus (especially type I) or other autoimmune deficiency, and the mass is firm and irregular, with a white, homogeneous solid section, and microscopically shows a lymphocytic infiltrate of mainly B lymphocytes in the lobules, periductal and perivascular areas (87–89). Lobular inflammation is more common in women, with widespread ductal inflammation, atrophy of the ducts and thickening of the basal lamina, and vasculitis involving mainly small and medium-sized vessels. It is associated with marked interlobular fibrosis, scar vitrification, epithelial fibroblasts, and lymphatic nodule formation with or without germinal centers (34, 90). Tomaszewski JE et al. suggest that epithelioid fibroblasts are unique to diabetic patient (91).

### 6.4 IgG4-related sclerosing mastitis (IgG4-RSM)

IgG4-related sclerosing mastitis is characterized by the formation of dense lymphoplasmacytic infiltrates, lymphoid follicle formation, occlusive phlebitis, extensive sclerosis, or fibrosis with at least a localized stellate distribution, with marked stromal sclerosis and loss of breast lobules (47, 72, 87). Immunohistochemical staining reveals large numbers of IgG4+ plasma cells and elevated IgG4 serum concentrations, but Goulabchand R et al. argue that in fact increased numbers of IgG4+ plasma cells are only part of the classical histological presentation and are not necessary for the diagnosis, nor does the diagnosis of GLM require consideration of the number of IgG4+ plasma cells or the IgG4:IgG ratio or the presence of other histological features of IgG4 -RSM (47, 92). Ogura K et al. have classified GLM into IgG4-associated and non-IgG4-associated subtypes (92). Interestingly Kong C et al. found that nipple invagination is a necessary basis for differentiating between IgG4-related and non-IgG4-related GLM, suggesting that the pathogenesis and immune mechanisms of the two diseases may be different and that the required treatment may be different (93).

### 6.5 Lupus mastitis

Lupus mastitis is characterized by lymphocytic lobular lipofuscinosis with plasma cell and hyaline fat necrosis (34). Lymphocytic infiltration may be nodular, diffuse, periductal and/or perifollicular and germinal center, and lymphocytic vasculitis is also common, involving mostly small and medium-sized vessels. Immunohistochemistry shows a mixed population of T and B lymphocytes, mainly CD3+ and CD4+ T cells mixed with CD20-positive B cells and polyclonal plasma cells (94).

### 6.6 Nodular disease and Wegener’s granulomatosis

When granuloma is present in conjunction with lymphocytic vasculitis, it needs to be differentiated from Wegener’s disease and nodular disease. Both nodular disease and Wegener’s granulomatosis affect mainly small and medium-sized vessels, the lesions are not lobularly centered, and both have necrotizing vasculitis and thrombosis, with a few cases of necrosis. Wegener’s disease is characterized by necrotizing vasculitis and granulomatous inflammation, mainly affecting the upper and lower respiratory tract and the kidneys (47). Nodular disease results in well-defined epithelioid nodules with microscopic lymphocytic infiltration in the vessel wall, but without neutrophil infiltration, and rarely vasculitis and fatty necrosis (25). Breast nodular disease is often secondary to generalized nodular disease, invading the dermis, with microscopic clusters of epithelioid cells of variable size, rarely extending into the subcutaneous tissue, and without neutrophil infiltration (39).

### 6.7 Plasma cell mastitis/periductal mastitis (PDM)/mammary duct ectasia (MDE)

Plasma cell mastitis is a late stage in the development of ductal dilatation of the breast or accompanies it, but is not an inevitable part of it. It mainly invades the large ducts behind the areola (especially within the 2 cm ring of the areola) (95). The affected ducts are highly dilated and the duct lumen contains secretions, exfoliated epithelium and foamy histiocytes. The duct wall is fibrotic and thickened, with atrophy of the epithelial cells of the duct wall. The duct is surrounded by a large infiltrate of diffuse lymphocytic plasma cells and other inflammatory cells, with a predominance of plasma cell infiltration. The ductal or lobular structures are frequently obscured or distorted (29, 34, 96). Necrosis may occur in the later stages and the masses are often interspersed with lipid-like (pimple-like) material after they have broken down (25). The mass is often interspersed with lipid-like material after rupture. Although GLM and ductal dilatation are pathologically distinct, they can occur together, and PDM can also present with granulomas and chronic purulent inflammation (75). The two can be difficult to distinguish when GLM is associated with dilated ducts and high plasma cell infiltration. The fusion lesions are mainly between GLM lesions, but fusion between ductal dilatation and GLM is rare (51). Cholesterol crystals and calcifications may also be present in MDE and are not statistically different from GLM (46).

### 6.8 Summary and outlook

Granulomatous lobular mastitis is a non-caseating granulomatous inflammatory disease occurring in the lobules of the breast. Granulomas are composed of epithelioid cells and multinucleated giant cells with an infiltration of inflammatory cells such as lymphocytes, neutrophils, plasma cells, and eosinophils, and as their features can be masked by focal fusion and abscess formation, as much breast tissue as possible should be selected to aid in the diagnosis. CNGM in the center of a granuloma containing neutrophils surrounded by lipid vesicles with Gram-positive bacilli visible in the vesicles is currently considered a subtype of GLM and its association with Corynebacterium is a current hot topic of research. However, recent studies have also tended to suggest that CNGM is a later stage of the GLM, a view that still needs to be confirmed. Our next step will be to conduct clinical studies based on this. We believe that the significance of pathology, in addition to diagnostic and differential diagnosis, can also contribute to the understanding of the disease by explaining its pathogenesis at the cytological level. Granulomatous lobular mastitis has been reported for the first time since 1972, and its incidence has gradually increased in recent years. Although there are many hypothese, the pathogenesis of granulomatous mastitis has not yet been fully established. In addition, GLM not only needs to be differentiated from breast cancer, but may also be combined with breast cancer and is a risk factor for breast cancer, which requires clinicians to spend effort on the diagnostic step of the disease. The relationship between inflammatory diseases of the breast, such as granulomatous lobular mastitis, and breast cancer needs to be further investigated. At present, animal studies of granulomatous mastitis have a high failure rate due to modeling difficulties, and are mostly clinical studies. In the future, there is a need not only for prospective clinical trials incorporating more samples, but also for collaborative imaging, testing and pathology contracts.

## Author contributions

LC: Writing – original draft. CS: Writing – review & editing. JG: Writing – review & editing. XZ: Writing – review & editing. SL: Writing – review & editing.
